# Integration of GWAS and cerebrospinal fluid proteogenomics identifies proteins for AD and shared dysregulated mechanisms in other neurological traits

**DOI:** 10.1002/alz.095164

**Published:** 2025-01-09

**Authors:** Daniel Western, Chengran Yang, Jigyasha Timsina, Priyanka Gorijala, Menghan Liu, Agustin Ruiz, Marta Marquié, Mercè Boada, Itziar de Rojas, Jarod Rutledge, Hamilton Se‐Hwee Oh, Edward N. Wilson, Yann Le Guen, Michael D Greicius, Tony Wyss‐Coray, Ignacio Alvarez, Miquel Aguilar, Pau Pastor, Chloe Robins, David J. Pulford, Yun Ju Sung, Carlos Cruchaga

**Affiliations:** ^1^ NeuroGenomics & Informatics Center, Washington University School of Medicine, St. Louis, MO USA; ^2^ Department of Psychiatry, Washington University School of Medicine, St. Louis, MO USA; ^3^ Networking Research Center on Neurodegenerative Diseases (CIBERNED), Instituto de Salud Carlos III, Madrid Spain; ^4^ Research Center and Memory Clinic, Fundació ACE Institut Català de Neurociències Aplicades ‐ Universitat Internacional de Catalunya (UIC), Barcelona Spain; ^5^ Network Center for Biomedical Research in Neurodegenerative Diseases (CIBERNED), Madrid Spain; ^6^ Research Center and Memory Clinic, ACE Alzheimer Center Barcelona, Universitat Internacional de Catalunya, Barcelona Spain; ^7^ Wu Tsai Neurosciences Institute, Stanford University, Stanford, CA USA; ^8^ Stanford University, Stanford, CA USA; ^9^ Stanford University, School of Medicine, Stanford, CA USA; ^10^ Memory Disorders Unit, Department of Neurology, Hospital Universitari Mutua de Terrassa, Terrassa, Barcelona, Terrassa Spain; ^11^ Unit of Neurodegenerative diseases, University Hospital Germans Trias i Pujol, Badalona, Barcelona Spain; ^12^ Human Genetics & Genomics, Research Technologies, GSK, Upper Providence, PA USA; ^13^ Human Genetics & Genomics, Research Technologies, GSK, Stevenage, Hertfordshire United Kingdom; ^14^ Division of Biostatistics, Washington University School of Medicine, St. Louis, MO USA; ^15^ Hope Center for Neurological Disorders, Washington University School of Medicine, St. Louis, MO USA

## Abstract

**Background:**

Cerebrospinal fluid (CSF) is a valuable resource for the study and diagnosis of neurological diseases, but few studies have comprehensively characterized the genetic determinants of CSF protein levels that may contribute to the development of disease. These quantitative trait loci (QTL) have proven vital to identifying candidate genes for disease treatment and monitoring. Here, we utilize our largest‐to‐date CSF protein QTL atlas to prioritize potentially causal proteins for 14 neurological traits and examine the unique and overlapping disease mechanisms observed using CSF proteins.

**Method:**

We generated the largest CSF proteogenomic dataset (3,506 samples, 6,361 proteins) to date. We performed proteome‐wide association study (PWAS), colocalization, and Mendelian Randomization (MR) to identify potentially causal proteins for 14 neurodegenerative and psychiatric diseases or disorders. We then performed pathway analysis to identify dysregulated mechanisms underlying disease and used RHOGE to assess the overlap of proteogenomic profiles between diseases. We are repeating these analyses using blood plasma to assess overlap with a more accessible tissue.

**Result:**

Across the 14 diseases, we identified 171 protein‐disease associations in CSF through at least two of PWAS, COLOC, and MR. For Alzheimer’s disease (AD), we identified 38 proteins enriched in immune‐relevant pathways (TREM2, CD33, IL34) and lysosomal function (GRN, CLN5, TMEM106B). Many proteins were found for schizophrenia (38), multiple sclerosis (27), and bipolar disorder (22), among others. We identified negative genetic correlations between PD and neuroticism and between anorexia nervosa and most other psychiatric traits. We observed limited overlap between CSF and plasma pQTLs (1,225/4,767 colocalize; 25.7%) with a moderate correlation in pQTL effect size (*R =* 0.576). Disease‐specific analyses in plasma are ongoing.

**Conclusion:**

We have identified numerous novel associations for neurological diseases that may represent therapeutic or biomarker targets in disease. We highlighted substantial differences in proteogenomic regulation between CSF and plasma, demonstrating the importance of investigating novel tissues in genetic studies. Through the integration of analyses from both tissues (ongoing), we will uncover disease‐relevant changes specific to neurological tissues or shared between tissues that may allow for diagnosis or prioritization of individuals for treatment based solely on proteomics generated from accessible tissues.

